# RpoE Facilitates Stress-Resistance, Invasion, and Pathogenicity of *Escherichia coli* K1

**DOI:** 10.3390/microorganisms10050879

**Published:** 2022-04-22

**Authors:** Yu Fan, Jing Bai, Daoyi Xi, Bin Yang

**Affiliations:** 1 The Key Laboratory of Molecular Microbiology and Technology, Ministry of Education, Nankai University, Tianjin 300457, China; 1120170077@mail.nankai.edu.cn (Y.F.); bj1551880084@163.com (J.B.); 2 Tianjin Key Laboratory of Microbial Functional Genomics, TEDA Institute of Biological Sciences and Biotechnology, Nankai University, Tianjin 300457, China; 3 Tianjin Institute of Industrial Biotechnology, Chinese Academy of Sciences, Tianjin 300308, China; daoyixi@126.com; 4 Key Laboratory of Systems Microbial Biotechnology, Chinese Academy of Sciences, Tianjin 300308, China

**Keywords:** HBMECs, bacteremia, meningitis, *E. coli* K1, RpoE, antibacterial peptides

## Abstract

*Escherichia coli* K1 is the most common Gram-negative bacterium that causes neonatal meningitis; thus, a better understanding of its pathogenic molecular mechanisms is critical. However, the mechanisms by which *E. coli* K1 senses the signals of the host and expresses toxins for survival are poorly understood. As an extracytoplasmic function sigma factor, RpoE controls a wide range of pathogenesis-associated pathways in response to environmental stress. We found that the Δ*rpoE* mutant strain reduced the binding and invasion rate in human brain microvascular endothelial cells (HBMECs) in vitro, level of bacteremia, and percentage of meningitis in vivo. To confirm the direct targets of RpoE in vivo, we performed qRT-PCR and ChIP-qPCR on known toxic genes. RpoE was found to regulate pathogenic target genes, namely, *ompA*, *cnf1*, *fimB*, *ibeA*, *kpsM*, and *kpsF* directly and *fimA*, *aslA*, and *traJ* indirectly. The expression of these genes was upregulated when *E. coli* K1 was cultured with antibacterial peptides, whereas remained unchanged in the presence of the Δ*rpoE* mutant strain. Moreover, RpoE reduced IL-6 and IL-8 levels in *E. coli* K1-infected HBMECs. Altogether, these findings demonstrate that RpoE mediates the host adaptation capacity of *E. coli* K1 via a regulatory mechanism on virulence factors.

## 1. Introduction

During the first few postnatal months of life, *Escherichia coli* K1 is one of the primary causative agents of neonatal bacterial meningitis, which is associated with high mortality and morbidity worldwide every year [1,2,3]. Clinical data show that *E. coli* has emerged as a common cause of early onset sepsis and meningitis among very low birth weight infants, which can cause serious complications, with a morbidity rate of 1.04% and mortality rate of 35.3% [4]. Empiric treatment often incorporates multiple antibiotics, such as ampicillin and cefotaxime, or similar third-generation cephalosporins, while anti-inflammatory agents may be added to prevent neurologic sequelae, such as hearing loss [5,6,7]. Despite timely treatment, many patients experience long-term neurological complications. To date, our incomplete understanding of the pathogenesis of this disease has hindered our ability to decrease its mortality and morbidity rates.

The pathology and clinical symptoms of *E. coli* K1 infection are sepsis and meningitis [8,9]. When *E. coli* K1 crosses the blood–brain barrier in vivo, it requires a threshold level of bacteremia to invade the brain microvascular endothelial cells (HBMECs) and arrive at the niche cerebrospinal fluid (CSF) [10]. Previous studies have shown that high-degree bacteremia (e.g., >10^5^ CFU/mL of neonatal animal blood [10]) significantly promotes the development of meningitis compared to low-degree bacteremia (e.g., <10^4^ CFU/mL of neonatal animal blood [10]) in laboratory animals by allowing *E. coli* K1 to escape host defenses and cause meningitis. *E. coli* K1 crosses the blood–brain barrier (BBB) through a transcytosis process without disrupting the tight junction in the BBB. Binding to and invasion of HBMECs by *E. coli* K1 are the prerequisites for successful penetration into the brain parenchyma [11]. During infection, many bacterial factor toxins, adhesins (S fimbriae, Type I fimbriae, and nonfimbrial adhesins) [12,13,14,15], protein secretion systems (T6SSs) [16], invasins (IbeA and CNF1) [17,18,19,20,21], and K1 capsules (KpsMT and NeuDB) [10,22,23,24] help *E. coli* K1 survive the host innate immunity, thereby facilitating the binding, invasion and crossing of brain microvascular endothelial cells.

RpoE, whose molecular weight is 24 kDa, is an alternative sigma factor that regulates the transcription of various genes in *E. coli* in response to cell envelope stress and plays essential roles in the pathogenesis of numerous bacterial pathogens, including *E. coli* [25,26], *Salmonella* [27,28,29], *Vibrio cholerae* [30], *Vibrio alginolyticus* [31,32], and *Treponema pallidum* [33]. In *E. coli*, *rpoE* is a component of a five-gene operon (*rseD-rpoE-rseA-rseB-rseC*), which also includes genes encoding the anti-sigma factor RseA, the negative regulator RseB, a positive modulator RseC, and the leader peptide RseD. Further, RpoE controls the expression of conserved genes that are functionally related to membrane integrity (e.g., *ompA*, *ompC*, and *ompF*) [34]; thus, it is essential for bacterial survival.

However, the specific role of RpoE in mediating *E. coli* K1 virulence has not been elucidated. In this study, we investigated the involvement of RpoE in the virulence of *E. coli* K1. The deletion of *rpoE* reduced the expression of many virulence factors and the virulence of *E. coli* K1 in vitro and in vivo. Moreover, we found that RpoE is induced by antibacterial peptides present in the blood and that it directly regulates many virulence genes. This is the first report to describe the regulation mechanism of RpoE in *E. coli* K1.

## 2. Materials and Methods

### 2.1. Ethics Statement

All animal experiments were conducted in accordance with the criteria specified in the Guide for the Nursing and Use of Laboratory Animals. Animal research procedures were approved by the Institutional Animal Care Committee at Nankai University and Tianjin Institute of Pharmaceutical Research New Drug Evaluation (IACUC 2016032102, 21 March 2016), Tianjin, China. Every effort was made to minimize animal suffering and reduce the number of animals used.

### 2.2. Bacterial Strains, Plasmids and Growth Conditions

The bacterial strains and plasmids used in the present study are listed in Supplementary Table S1. The oligonucleotide primers used in this study are listed in Supplementary Table S2. *E. coli* K1 RS218 was used as the WT strain. Mutant strains were generated by the λ Red recombinase system of pSim6 using primers carrying 50-bp homologous regions flanking the start and stop codons of the gene to be deleted, as previously described [35]. All strains were grown at 37 °C in brain-heart infusion (BHI) medium.

### 2.3. E. coli Binding and Invasion Assay in HBMECs

HBMECs were cultured in RPMI 1640 medium (Thermo Scientific, Waltham, MA, USA) supplemented with 10% fetal bovine serum (FBS, Hyclone, Marlborough, MA, USA), 10% Nu-serum (BD Biosciences, Franklin Lakes, NJ, USA), 2 mM glutamine, 1% MEM nonessential amino acids (Wako, Osaka, Japan), 1 × MEM vitamin (Sigma, Irvine, UK), 100 U/mL penicillin, 100 μg/mL streptomycin, and 1 mM sodium pyruvate (Thermo Scientific). *E. coli* K1 strains were resuscitated from −80 °C, cultured overnight, and then subcultured in fresh BHI medium at a dilution of 1:100 until strains were grown to the exponential phase at an OD_600_ of 0.6. Bacteria were collected by centrifugation and resuspended in RPMI 1640 medium containing 10% FBS. HBMECs infected at a multiplicity of infection (MOI) of 100 were incubated for 1.5 h in a cell incubator. The HBMECs were then washed with phosphate-buffered saline (PBS) to remove unbound bacteria and incubated with new medium containing gentamicin (100 mg/mL) for 1 h to kill extracellular *E. coli*. HBMECs were washed, lysed with 0.5% triton X-100, and plated on Luria broth (LB) agar plates for the determination of CFUs. A binding assay was performed similarly to the invasion assay except that the gentamicin treatment step was omitted. All experiments were conducted in duplicate and performed at least in triplicates.

### 2.4. Animal Model of E. coli Bacteremia and Hematogenous Meningitis

*E. coli* bacteremia was induced in 14-day-old BalB/C mice (Beijing Vital River Laboratory Animal Technology Co., Beijing, China). All mice were injected with 1 × 10^7^ CFU of *E. coli* in the exponential phase at an OD_600_ of 0.6 via the tail vein. At 4 h after bacterial inoculation, blood specimens were obtained for quantitative cultures and RNA extraction. For the determination of CFUs, bacteria in the samples from the blood were assessed by plating on LB agar plates. For RNA extraction, blood samples were processed using TRIzol reagent (Invitrogen, Waltham, MA, USA). For hematogenous meningitis, all 14-day-old BalB/C mice were injected with 1 × 10^6^ CFU of *E. coli* in the exponential phase at an OD_600_ of 0.6 via the tail vein. At 4 h after bacterial inoculation, CSF specimens were obtained to enumerate CFUs.

### 2.5. qRT–PCR

For the antibacterial peptide assay, the bacteria were cultured in M9 medium with or without LL-37 (Sigma) and incubated at 37 °C for 1–2 h. Blood or bacteria were pelleted by centrifugation. RNA samples were isolated using TRIzol (Invitrogen), reverse transcribed using the PrimeScript^TM^ RT reagent Kit (TaKaRa, Kusatsu, Japan), and processed for qRT–PCR. Each qRT–PCR was reacted in Power SYBR Green PCR Master Mix (Applied Biosystems, Waltham, MA, USA). The fold-change in the target gene relative to the housekeeping gene *dnaE* was determined using the 2^−^^ΔΔCt^ method. At least three biological replicates were used for each qRT–PCR analysis.

### 2.6. ChIP-qPCR

RS218 WT containing pBAD24-RpoE/FLAG was cultured in RPMI 1640 medium supplemented with 0.1% arabinose until the exponential phase and then treated with 1% formaldehyde for Cross-linking, then stopped by adding 125 mM glycine. The bacterial pellets were washed with PBS, resuspended in immunoprecipitation buffer (IP; 50 mM pH 7.5 HEPES–KOH, 150 mM NaCl, 1 mM EDTA, 1% Triton X-100, 0.1% sodium deoxycholate, 0.1% SDS, and protease inhibitor cocktail (MCE)), and then subjected to sonication. The program was set to 30 cycles of a 5 s sonication followed by 10 s pauses (30% amplitude, 7.5 min total). Insoluble cellular debris was removed by centrifugation, and the supernatant was used as the input sample in the IP experiments. Both mock and IP samples were incubated with mouse IgG1 isotype control antibodies and anti-FLAG antibodies (Sigma), respectively, and then incubated with protein A beads (Life Technologies, Carlsbad, CA, USA) in IP buffer. Washing, crosslink reversal, and purification of the ChIP DNA were performed.

ChIP-qPCR was performed as described previously [36]. To measure the enrichment of potential RpoE-binding targets in the immunoprecipitated DNA samples, percent input and fold enrichment were performed using SYBR green mix. The relative target levels were calculated using the ΔCt method. *lacZ* was used as a negative control, with specific details referring to the ChIP Analysis [36]. The results were reported as the average enrichment of three biological replicates.

### 2.7. ELISA Analyses

HBMECs (1 × 10^6^) were grown in 6-well plates and incubated with 50 g/mL Hcp family proteins per well for 6 h at 37 °C in a 5% CO_2_ incubator. The supernatants were collected and centrifuged to remove cell debris. The cytokine levels in the supernatant were determined using the Human IL-6 and IL-8 DuoSet^®^ ELISA detection kits (R&D Systems, Minneapolis, MN, USA).

### 2.8. Fluorescent Actin Staining

Fluorescent actin staining (FAS) was performed as described previously [37]. Briefly, overnight bacteria were subcultured in BHI and incubated until an OD_600_ of 0.6 was obtained. Then, the bacteria were diluted on coverslips to infect HBMECs at MOI = 100 in the exponential phase at an OD_600_ of 0.6. After incubation, the coverslips were washed and fixed with formaldehyde; the cells were permeabilized with 0.1% Triton-X and stained with AF647-labeled phalloidin to visualize the actin filaments. *E. coli* was stained with FITC labeled anti-*E. coli* K and O antigen antibody (Abcam, Cambridge, UK). HBMEC nuclei were stained with 6-diamidino-2-phenylindol (DAPI). Binding and invasion assays on each cell line were performed, with three slides per experiment.

### 2.9. Brain Slice Immunofluorescence

Fourteen-day-old BALB/c mice were injected with 1 × 10^7^ CFU of WT or Δ*rpoE* via the tail vein. Brain specimens were prepared on a Leica Cm1950 cryostat platform (Leica Microsystems, Buffalo Grove, IL, USA). The slices were fixed, and blocked. *E. coli* was stained with FITC-conjugated Anti-*E. coli* O and K antigen antibody (Abcam). DAPI was stained for nucleus visualization. Slices were then observed using a Zeiss LSM800 confocal microscope (Zeiss, Oberkochen, Germany). Each experiment was repeated at least three times. 

### 2.10. Capsule Staining

Capsular staining [38] was performed using a capsular staining kit (Solarbio, Beijing, China). Staining was performed according to the manufacturer’s instructions. The capsule and colony morphology were observed via optical microscopy (Leica DM2500).

### 2.11. Growth Assay

To determine the growth curve of each strain, overnight cultures were diluted to a 1:100 ratio in a flask containing 200 mL of BHI broth without antibiotics and incubated at 37 °C with shaking at 180 rpm. A 200 μL aliquot was added to a 96-well flat bottom microplate and incubated at 37 °C with shaking at high speed for 24 h. The absorbance at 600 nm was recorded. Experiments were independently performed three times.

### 2.12. Statistical Analysis

Statistical analyses were conducted using the GraphPad Prism software (v8.3.0). GraphPad Software, San Diego, CA, USA; https://www.graphpad.com/scientific-software/prism/ (accessed on 20 April 2022). The mean ± standard deviation values of three independent experiments are shown in the figures. Differences between two mean values were evaluated using a two-tailed Student’s *t*-test. Statistical significance was assessed using the two-sided Fisher exact test in the mouse experiments. Statistical significance was set at *p* < 0.05.

## 3. Results

### 3.1. rpoE Promotes E. coli K1 Binding and Invasion of HBMECs

**Figure 1:**
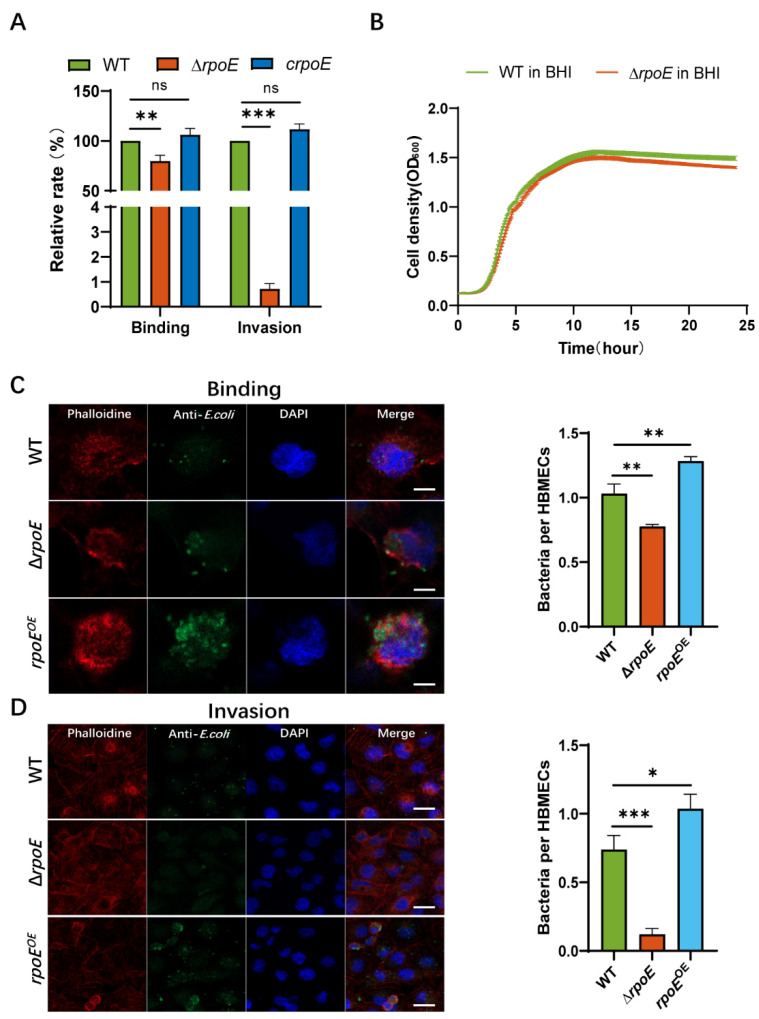
Deletion of *rpoE* reduces *Escherichia coli* K1 binding to and invasion of human brain microvascular endothelial cells (HBMECs). (**A**) The binding and invasion abilities of Δ*rpoE* and *crpoE* strains were calculated relative to those of the wild-type (WT) strain. (**B**) Growth of RS218 WT and Δ*rpoE* strains in brain-heart infusion (BHI) medium. (**C**) Number of bacterial cells per each HBMEC determined in random fields of fluorescent actin staining (FAS) in a binding assay. Average of at least 50 cells is shown (left). Scale bar, 5 μm. (**D**) Number of bacterial cells per HBMEC determined in random fields of FAS in an invasion assay. Average of at least 50 cells is shown (left). Actin cytoskeleton (red) and nuclei (blue) of the HBMECs, and bacterium (green) are shown. Scale bar, 20 μm. * *p* < 0.05, ** *p* < 0.01, *** *p* < 0.001 by Student’s *t*-test. ns, not significant.

To determine the role of *rpoE* in the invasion of HBMEC by *E. coli* K1, an isogenic in-frame deletion mutant of *rpoE* was constructed. The regulatory gene *rpoE* is required for the binding and invasion of HBMECs by *E. coli* K1 strain RS218, which causes neonatal meningitis. To examine the virulence phenotype of the *rpoE* deletion mutant, we performed a comparative study of the binding and invasion capability of RS218 WT (parent strain) and Δ*rpoE*. The binding and invasion rates of *rpoE* deletion in HBMECs reduced by 20.2% and 99.28%, respectively, compared with those in the WT strain (Figure 1A). The binding and invasion rates of the complemented strain (*crpoE*) were comparable to that of the WT strain (Figure 1A). Immunofluorescence microscopy confirmed the effect of *rpoE* deletion on *E. coli* K1 binding and HBMEC invasion. Consistent with the findings of the in vitro assay, FAS shows less binding by the Δ*rpoE* mutant and invasion of HBMECs, compared to RS218 WT and the *rpoE*-overexpression strain (*rpoE*^OE^) induces higher binding and invasion rates than that of the WT strain during HBMECs infection (Figure 1C,D). The growth curve of the Δ*rpoE* mutant is similar to that of the RS218 WT strain (Figure 1B), indicating that the decrease in binding and invasion is not due to the observed growth rates. 

### 3.2. RpoE Is Involved in the Survival of E. coli K1 in Blood and Meningitis In Vivo

**Figure 2:**
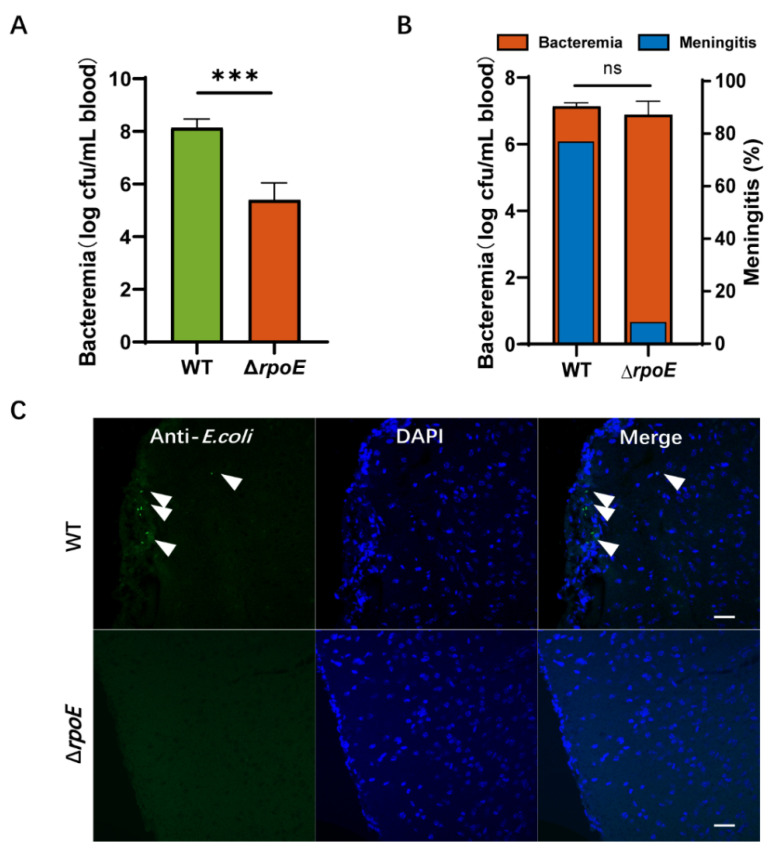
Deletion of *rpoE* reduces RS218 survival in the blood and penetration of the blood–brain barrier (BBB). (**A**) Magnitude of bacteremia in BALB/c mice. Bacterial counts in the blood (log CFU/mL) were determined. (**B**) Blood samples were collected for bacteremia measurement, and cerebrospinal fluid (CSF) was collected and cultured to assess the passage of bacteria through the BBB. (**C**) The brains were collected after the mice were intravenously inoculated with bacteria and were sectioned for immunofluorescent staining. *E. coli* K1 (green) and nuclei (blue) are shown. Arrows indicate bacteria. Scale bar, 20 μm. The data represent the mean ± standard deviation from three independent experiments performed in triplicate. *** *p* < 0.001 by Student’s *t*-test, ns, not significant.

To investigate the role of *rpoE* in *E. coli* K1 infection, we examined the ability of the RS218 WT and Δ*rpoE* strains to survive in the blood of BALB/c mice. Each animal received 1 × 10^7^ CFU of RS218 WT and Δ*rpoE* strains via tail vein injection to induce meningitis. Then, blood specimens were collected and cultured for divergent levels of bacteremia (WT, 8.14 ± 0.10 mean log CFU/mL of blood; Δ*rpoE*, 5.39 ± 0.40 mean log CFU/mL of blood, *p* < 0.001, Figure 2A), suggesting that *rpoE* expression plays a role in promoting *E. coli* K1 proliferation in mouse blood.

CSF was collected and cultured to illustrate the onset of bacterial infection in the central nervous system (CNS). The rate of meningitis occurrence, defined as a positive CSF culture with meningeal inflammation, is reduced to 8.33% by the Δ*rpoE* strain (*n* = 12), which is significantly (*p* = 0.001 by two-sided Fisher exact test) lower than the 76.92% by the parent strain RS218 WT (*n* = 13, Figure 2B). In the mice with *E. coli* meningitis, we found the presence of WT strains in the brain’s parenchyma (5.0 ± 1.4 bacteria/slice, Figure 2C), but Δ*rpoE* was not found. These data suggest that the *rpoE* gene contributes to penetration of the blood–brain barrier in vivo.

### 3.3. Identification of the Virulence Factors That Are Regulated by RpoE

**Figure 3:**
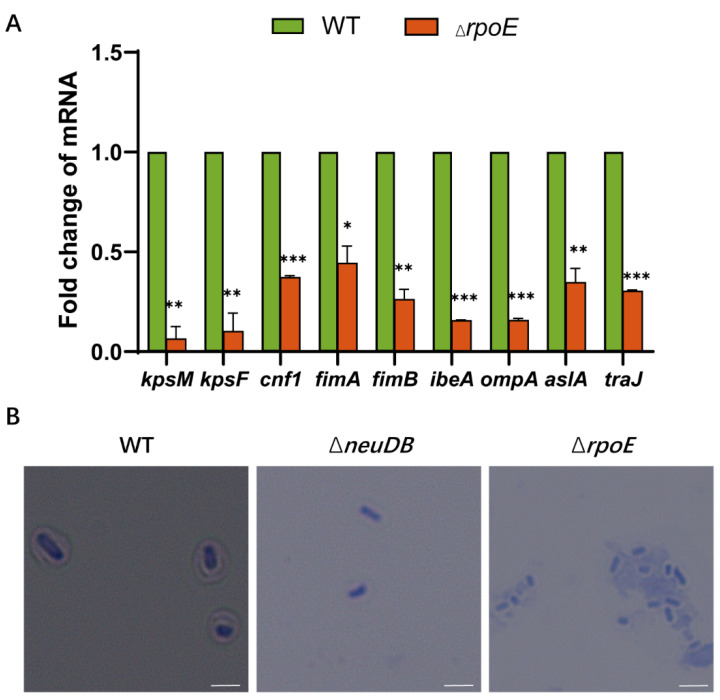
RpoE is a positive virulence regulator of *E. coli* K1. (**A**) Fold changes in genes transcription in the *rpoE* mutant strain relative to their expression in the WT strain are shown. (**B**) Capsule morphology Scale bar, 3 μm. Capsule staining of WT, Δ*neuDB*, and Δ*rpoE* strains * *p* < 0.05; ** *p* < 0.01; *** *p* < 0.001.

To investigate the regulatory network of RpoE, qRT-PCR analysis was performed. Bacterial RNA was extracted from the blood of mice infected with RS218 WT and *rpoE* mutant strains via tail vein injection and used for qRT–PCR. *E. coli* K1 replication in blood and invasion of HBMECs depends on various outer membrane proteins, fimbriae, and effectors, such as K1 capsule (*kpsM*, *kpsF*), *cnf1*, *fimA*, *fimB*, *ibeA*, *ompA*, *aslA*, and *traJ*, the levels of which decreased by 0.064-, 0.101-, 0.371-, 0.442-, 0.261-, 0.154-, 0.156-, 0.346-, and 0.302-fold (Figure 3A), respectively, in *rpoE* mutant. We speculate RpoE can directly upregulate the yield of the K1 capsule by activating the expression *kpsM* and *kpsF*, which are the first gene of region 2–3 and region 1 transcriptional units, respectively [39]. Capsule staining shows K1 capsule presence in the WT strain but not in the Δ*neuDB* and Δ*rpoE* mutant strains (Figure 3B).

**Figure 4:**
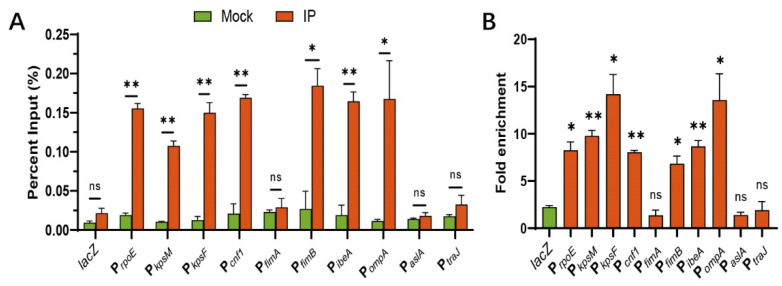
Virulence factors are directly regulated by RpoE in vivo. (**A**) Percent input of the promoter region of virulence factors in RpoE-ChIP samples. (**B**) Fold enrichment of the promoter region of virulence factors in RpoE-ChIP samples. * *p* < 0.05; ** *p* < 0.01; ns, not significant by Student’s *t*-test.

ChIP-qPCR was performed to identify which toxic genes could be directly regulated by RpoE. RpoE binds to a highly conserved ‘AAC’ motif and a clustering of ‘CGT’ trinucleotides in the 35 region and 10 region, respectively [40], and activates its own promoter in response to cytoplasmic stress [34]. Therefore, we used *rpoE* as a positive control and a (GC)-rich gene *lacZ* as a negative control for the ChIP assay. ChIP-qPCR analysis revealed that P*_rpoE_*, P*_kpsM_*, P*_kpsF_*, P*_cnf1_*, P*_fimB_*, P*_ibeA_*, and P*_ompA_*, were enriched 8.25-, 9.77-, 14.19-, 8.05-, 6.8-, 8.66-, and 13.55-fold, respectively, in IP-ChIP samples compared to those of mock ChIP samples (Figure 4A,B), confirming the binding of virulence genes in vivo. In contrast, the fold enrichment of *lacZ*, P*_fimA_*, P*_aslA_*, and P*_traJ_* does not differ between the IP-ChIP and mock-ChIP samples (Figure 4A,B). These results demonstrate that RpoE influences the expression of many virulence genes through direct or indirect regulation in vivo.

### 3.4. The Expression of rpoE Is Upregulated after the Infection of E. coli K1 In Vitro and In Vivo

**Figure 5:**
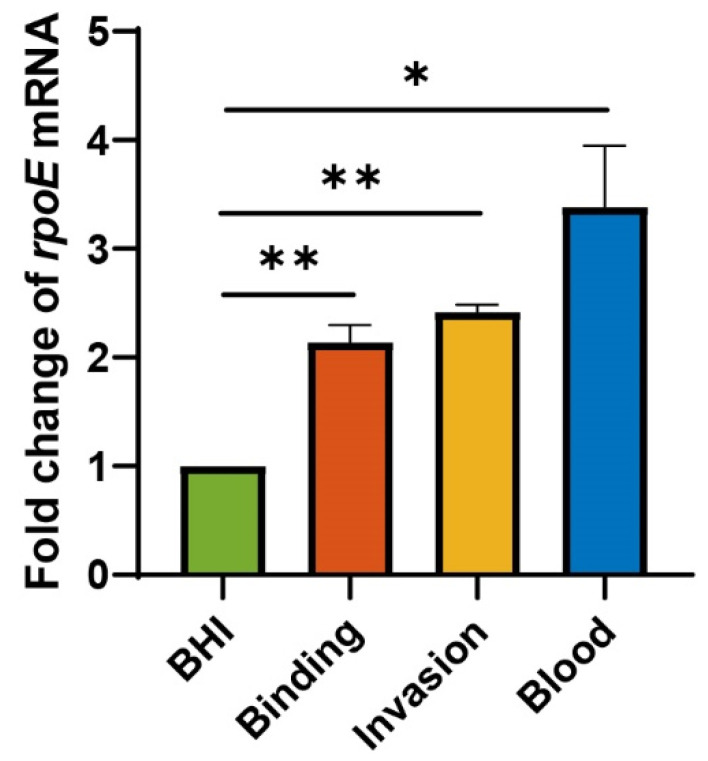
*rpoE* transcriptional levels are upregulated upon *E. coli* K1 infection in vitro and in vivo. * *p* < 0.05; ** *p* < 0.01 by Student’s *t*-test. Data are presented as the mean ± standard deviation; *n* = 3.

The transcriptional levels of *rpoE* vary during the diffident infectious phase. The expression of the *rpoE* gene increases the binding and invasion 2.141- and 2.418-fold, respectively (Figure 5), compared with that cultured in BHI. These data suggest that *rpoE* needs more expression to enhance the virulence in *E. coli* K1 binding and invasion of HBMECs. As the WT strain was able to form high-level bacteremia, we tested the expression of *rpoE* in blood compared to that in BHI (Figure 5). The expression level of *rpoE* was 3.385-fold higher in blood than that in the BHI medium. The above results illustrate that some signals in serum of PMI 1640 medium or blood activate the expression of *rpoE*.

### 3.5. RpoE Is Essential for the Cationic Antimicrobial Peptide-Dependent Expression of the Virulence Factors

**Figure 6:**
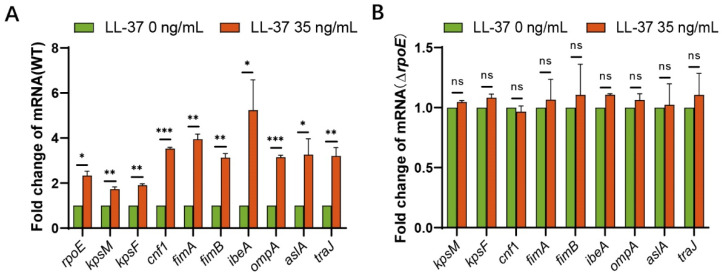
RpoE enhances the expression of virulence factors induced by LL-37. (**A**) Fold change in the transcriptional levels of toxic genes in the WT strain cultured with LL-37. (**B**) Fold change in the transcriptional levels of toxic genes in the Δ*rpoE* mutant strain cultured with LL-37. * *p* < 0.05; ** *p* < 0.01; *** *p* < 0.001; ns, not significant by Student’s *t*-test.

The serum concentrations of LL-37 in patients infected with *Enterobacteriaceae* were up to 86.12 ng/mL [41], but the concentration of LL-37 in a healthy infant was 0.3 ng/mL [42]. RpoE plays a necessary role in responding to cell envelope stresses such as a cationic antimicrobial peptide (AMP) [43]. The serum mean concentrations of LL-37 in mice with meningitis was 35 ng/mL (data is not published). To further investigate the molecular mechanism underlying RpoE-mediation, the regulation of virulence gene expression was measured using qRT–PCR with the cationic antimicrobial peptide LL-37 (35 ng/mL). Total RNA obtained from the WT or Δ*rpoE* cells was cultured in the M9 medium with or without LL-37. The expression of the *rpoE*, *kpsM*, *kpsF*, *cnf1*, *fimA*, *fimB*, *ibeA*, *ompA*, *aslA*, and *traJ* genes showed a 2.338-, 1.727-, 1.902-, 3.532-, 3.953-, 3.131-, 5.237-, 3.149-, 3.261-, and 3.320-fold increase with LL-37, respectively, compared to that in cultures without LL-37 (Figure 6A). Gene induction by LL-37 was abolished in the *rpoE* mutant strain (Figure 6B). The above results show that toxin induction by LL-37 requires RpoE.

### 3.6. RpoE Attenuates Inflammation of HBMECs Caused by E. coli K1

**Figure 7:**
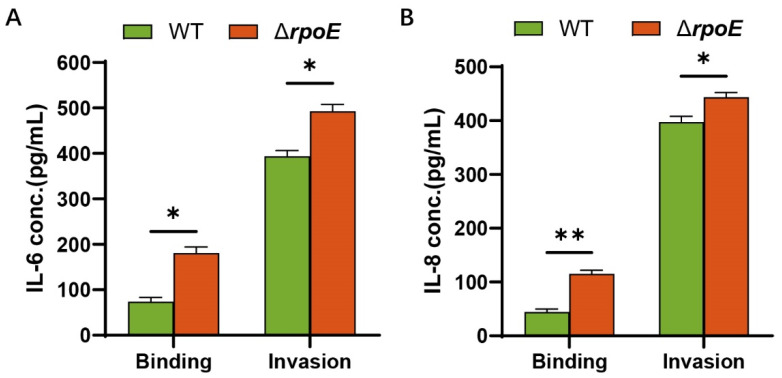
Cytokine release by HBMECs incubated with *E. coli* K1. (**A**) The levels of IL-6 in the culture supernatants of HBMECs that infected WT and Δ*rpoE*. (**B**) The levels of IL-8 in the culture supernatants of HBMECs that infected WT and Δ*rpoE*. Data were obtained from three independent experiments and analyzed using Student’s *t*-test. * *p* < 0.05, ** *p* < 0.01.

Since increased levels of cytokines occur in bacterial meningitis [16] and *rpoE* mutation attenuates the ability of *E. coli* K1 to cross the BBB, we investigated the role of RpoE in cytokine and chemokine secretion in HBMECs. Compared to those of the WT strain, significantly higher levels of IL-6 and IL-8 are released after the binding of the Δ*rpoE* strain to HBMECs (Figure 7A). After *E. coli* K1 invasion in HBMECs, the Δ*rpoE* strain caused significant increases in cytokine and chemokine release in HBMECs compared to those caused by the WT strain (Figure 7B). These results illustrate that RpoE attributes to attenuating inflammation of HBMECs caused by *E. coli* K1.

## 4. Discussion

The results of the present study revealed that RpoE regulates additional biological pathways, including those related to the capsule assembly, fimbria expression, and cell necrosis factor biogenesis, which were compared to the main biological pathways, including those for lipopolysaccharide biogenesis and outer membrane porin assembly [34,44]. There were significant differences associated with targets of RpoE between the results of our study based on *E. coli* K1 and those previously validated in *E. coli* K12. In this study, we established that RpoE promotes high-level bacteremia and meningitis associated with *E. coli* K1. RpoE upregulated the expression of toxic genes such as *cnf1*, *fimB*, *ibeA*, *ompA*, *kpsM*, and *kpsF* directly. Except for *ompA*, the rest were verified to be new target genes of RpoE. Meanwhile, RpoE indirectly upregulated the expression of toxic genes such as *fimA*, *aslA*, and *traJ*.

In response to environmental stress in *Salmonella*-containing vacuoles, RpoE upregulates the expression of several genes, including *htrA*, which is required for survival in *Salmonella typhimurium* intramacrophage [28]. The *rpoE*-mutant of *Salmonella* strain had reduced survival and infirmity to oxidative stress [28]. In contrast to the parental *Vibrio cholerae* in an infant mouse model, the lethality of the *rpoE* mutant was attenuated significantly, with a decreasing colonization ability to the intestine [30]. The increased expression level of RpoE was observed in the WT *E. coli* LF82 strain at high osmolarity, and the overexpression of RpoE in the Δ*ompR* isogenic mutant restored the phenotype of the WT strain [26]. The *rpoE* mutant *Vibrio alginolyticus* was incapacitated to adapt to environmental and host stresses, indicating that *rpoE* is critical for the activation of virulence genes in response to temperature [31]. To investigate whether RpoE is temperature-dependent with respect to the regulation of virulence in *E. coli* K1, qRT-PCR analysis was performed. However, the expression of *kpsM* and *kpsF* was not different between WT and *rpoE* mutant strains at various temperatures (Supplementary Figure S1), indicating that RpoE cannot regulate virulence in response to temperature in *E. coli* K1.

*Vibrio cholerae* El Tor was reported to resist the cationic antimicrobial peptide (AMP) of the intestinal tract, but the *rpoE* mutant of *V. cholerae* El Tor was more sensitive than the WT was to the cationic AMP (polymyxin B) [43,45,46]. Before *E. coli* K1 invades the BBB, it needs to replicate in the blood to induce high levels of bacteremia. Therefore, *E. coli* K1 can sense the cationic antimicrobial peptide of the blood via RpoE to upregulate the expression of many toxic genes to evade host immunity and cross the BBB finally. 

The Δ*rpoE* strain stimulated HBMECs to release more IL-6 and IL-8 than the WT strain did, which are crucial inflammatory mediators that elicit leukocyte infiltration in bacterial meningitis [16]. Recruitment of neutrophils is not conducive to the survival of bacteria. Meanwhile, the released cytokines disrupt the tight junctions of the BBB, causing a marked inflammatory response in the CNS [11,47]. This phenomenon may explain why *E. coli* K1 crossing the BBB is a key point in meningitis and central nervous system damage. RpoE-regulated target genes, such as K1 capsule, contribute to the evasion of host immune recognition by *E. coli* K1 [48,49].

**Figure 8:**
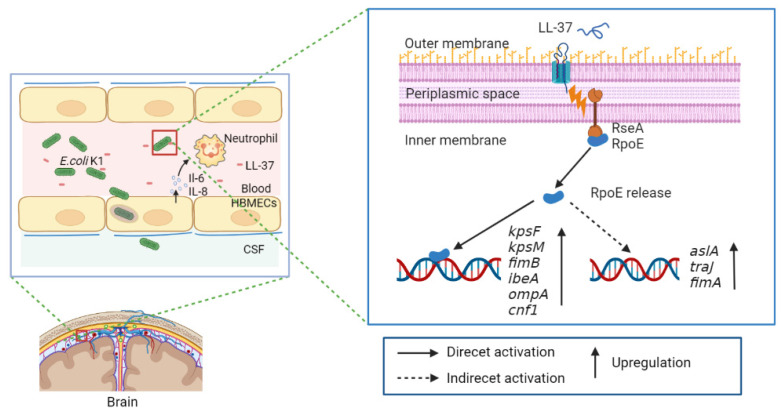
Model of the regulatory pathway of RpoE-mediated cationic antimicrobial peptide-dependent signaling in *E. coli* K1.

In conclusion, our data demonstrate that the extracytoplasmic function sigma factor RpoE contributes to the virulence of *E. coli* K1 by influencing the expression of known virulence factors (Figure 8). This regulatory mechanism is critical for the pathogenicity of *E. coli* K1 as the *rpoE* mutant results in significantly reduced virulence. The response to cationic AMP results in the activation of toxic genes via RpoE. Thus, our findings deepen our understanding of how *E. coli* K1 senses environmental signals to facilitate the overall adaptability of the pathogen.

## Data Availability

The data presented in this study are available in the Supplementary Material.

## Supplementary Materials

The following supporting information can be downloaded at: https://www.mdpi.com/article/10.3390/microorganisms10050879/s1, Figure S1. RpoE cannot respond to temperature to regulate the expression of kpsF and kpsM in *E. coli* K1; Table S1: Bacterial strains and plasmids used; Table S2: Primers used.
