# Supplementary material for: RpoE Facilitates Stress-Resistance, Invasion, and Pathogenicity of Escherichia coli K1
Source: Microorganisms. 2022 Apr 22;10(5):879. doi: 10.3390/microorganisms10050879 (PMC9147696; doi:10.3390/microorganisms10050879)
Supplement: Supplementary file 1 [file microorganisms-10-00879-s001.zip › microorganisms-1671089-supplementary.pdf]

microorganisms

Supplementary Materials

RpoE Facilitates Stress-Resistance, Invasion, and Pathogenicity of *Escherichia coli* K1

Yu Fan [1,2], Jing Bai [1,2], Daoyi Xi [3,4] and Bin Yang [1,2,*]

[1] The Key Laboratory of Molecular Microbiology and Technology, Ministry of Education, Nankai University, Tianjin 300457, China; 1120170077@mail.nankai.edu.cn (Y.F.); bj1551880084@163.com (J.B.)
[2] Tianjin Key Laboratory of Microbial Functional Genomics, TEDA Institute of Biological Sciences and Biotechnology, Nankai University, Tianjin 300457, China
[3] Tianjin Institute of Industrial Biotechnology, Chinese Academy of Sciences, Tianjin 300308, China; daoyixi@126.com
[4] Key Laboratory of Systems Microbial Biotechnology, Chinese Academy of Sciences, Tianjin 300308, China
* Correspondence: yangbin@nankai.edu.cn

Supplementary Materials

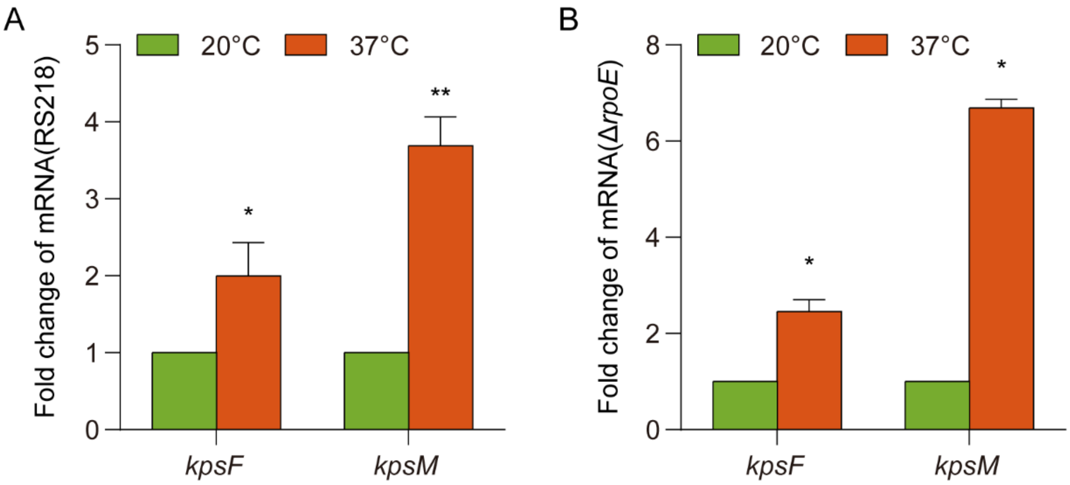

Figure S1. RpoE cannot respond to temperature to regulate the expression of *kpsF* and *kpsM* in *E. coli* K1. (**A**,**B**) qRT-PCR analysis of the transcription of *kpsF* and *kpsM* that were cultivated at different temperatures. Total RNA was isolated from the WT or Δ*rpoE* strains after they were grown in LB at different temperatures for 2 h. Data were obtained from three independent experiments and analyzed using Student's *t*-test. * $p < 0.05$, ** $p < 0.01$.

Table S1. Bacterial strains and plasmids used.

	Genotype or Description	Source
	Escherichia coli **K1 strains**	
WT	Wild-type *E. coli* K1 RS218	Our laboratory
Δ*rpoE*	WT strain *rpoE*::Cm; Cm^R	This study
c*rpoR*	Δ*rpoE* strain harboring plasmid pACYC184-RseD-RpoE-RseABC; Tc^R; complemented strain	This study
rpoE^{OE}	WT harboring plasmid pBAD-RpoE; Ap^R; overexpression strain	This study
**Plasmids**		
pKD3	For λ Red recombination; Cm^R	Our laboratory
pSim6	For generating mutant strains with λ Red recombinase system under a temperature-inducible promoter; Ap^R	Our laboratory
pBAD24	Expression vector; Ap^R	Our laboratory
pBAD-RpoE	pBAD24 carrying the WT *rpoE* gene; Ap^R	This study
pACYC184-RseD-RpoE-RseABC	pACYC184 carrying the RseD-RpoE-RseABC operon; Tc^R	This study

Table S2. Primers used.

Target Gene		Primer Sequence (5′–3′)
		Primers for the construction of the mutant strain *
rpoE	F	TAGCGCGTGGAAATTTGGTTTGGGGAGACTTTACCTCGGCATATGAATATCCTCCTTAG
	R	TCTGCATGCCTAATACCCTTATCCAGTATCCCGCTATCGGTGTAGGCTGGAGCTGCTTCG
		Primers for the identification of the mutant
rpoE	F	CTTCAGGCAGTTAAATGGGC
	R	GCATTGAGTCACGGATCAAG
		Primers for the construction of the clone strain
RpoE-Flag	F	TGGGCTAGCAGGAGGAATTCATGAGCGAGCAGTTAACGGAC
	R	CCGCCAAAACAGCCAAGCTTCTACTACTTGTCATCGTCATCCTTGTAATCGATGTCATGA TCTTTATAATCACCGTCATGGTCTTTGTAGTCACGCCTGATAAGCGGTTGAAC
RpoE-comp	F	CCCATGGACGTCACATGAATGTTCAGGG
	R	CCGGAATTCGTTCATACCGTATGGATTGTGG
		qRT-PCR primers
rpoE	F	AGGTTATTGCCATGCGTAA
	R	GCTGTATCGGATTGCTGTA
dnaE	F	GGCAATCGTATCGGTATCT
	R	GCTTCGTCAATATCCATCAC
ompA	F	ATCCAGAGCAGCCTGACCTTCC
	R	CAACAACATCGGTGACGCACAC
kpsF	F	ATGCTGATGCCGTGCTGGAAC
	R	GCAGGCGACGACCTAATGAACC
kpsM	F	AGTTCAGACTCACGCCTTCACT
	R	AGGTCCTGCCGATACTGCTTAA
fimA	F	AGCCGCTGTTGCCTTCTTGG
	R	CGCCTGGAACGGAATGGTGTT
aslA	F	TGGTCTGGACTGGATGCCTACT
	R	CCATCACGGATCGCCCATTCAT
cnf1	F	GAGGCGTTGATGGCTCAGGAA
	R	GCAGCAAGCAGACGACACTTTC
traJ	F	AGGCTCTTGGACATGGCTCTG
	R	ACAACCAATGTCCCTGGAGAAA

fimB	F	CACTGGAGATTCATCCGCACAT
	R	GCTGTCGTCCTCTGGCTCTATC
ibeA	F	CGTTAGGCGGTTCCTGGAGTAC
	R	CTGGAATTGACGCAACGCTTCA
		Primers for ChIP-qPCR
rpoE	F	ACGCATCTGTTTTTGTTTGTC
	R	GTTAAATGGGCATTTCTACACAG
lacZ	F	CACCGCCGAAAGGCGCGG
	R	CAGCCGCGCGGTACTGGAG
ompA	F	GATCTGCTCAATATTAACTCTACCG
	R	TGACGGAGTTCACACTTGTAAG
kpsF	F	CCTGCATATAAGCATGGACTGACC
	R	AGTTATGCCTGGCTATATCAGTG
kpsM	F	GCGGCTATTAAAAAGGTCAAACCG
	R	GCCATTTGATGATGTGATCCTAATCTC
fimA	F	GCGCGATGCTTTCCTCTATGAG
	R	CGACATGGGCAGTCGTTCTG
aslA	F	GGACAGAAATGATGCCTATGCGG
	R	CCACGTCACACTGGCTTTCC
cnf1	F	GGAGCATCTCCAGTGTTCC
	R	ATGGGGATATAATGCTGTTCTG
traJ	F	CTGAGATGGAGAGGTTCTTTCCAG
	R	ACCTTTATGCGGTTAATTGTCATC
fimB	F	ATGTTGGATTATTGCTAACCCAG
	R	ACTATGGGTCAGGAAGTTCC
ibeA	F	GTCTGTATTAGCATGATGTTGCTTG
	R	CGAGCGGGTTCCAGATAAAATTCC

* Primers were designed to harbor extensions homologous to ~40 bp (underlined) of the target gene. F, forward; R, reverse.
